# Ultra-fast cell counters based on microtubular waveguides

**DOI:** 10.1038/srep41584

**Published:** 2017-01-30

**Authors:** Cornelius S. Bausch, Christian Heyn, Wolfgang Hansen, Insa M. A. Wolf, Björn-Philipp Diercks, Andreas H. Guse, Robert H. Blick

**Affiliations:** 1 Institute of Nanostructure and Solid State Physics, University of Hamburg, Jungiusstraße 11c, Hamburg, Germany; 2 Center for Hybrid Nanostructures, University of Hamburg, Falkenried 88, Hamburg, Germany; 3 Calcium Signaling Group, Department of Biochemistry and Molecular Cell Biology, University Medical Center Hamburg-Eppendorf, Martinistraße 52, Hamburg, Germany

## Abstract

We present a radio-frequency impedance-based biosensor embedded inside a semiconductor microtube for the in-flow detection of single cells. An impedance-matched tank circuit and a tight wrapping of the electrodes around the sensing region, which creates a close, leakage current-free contact between cells and electrodes, yields a high signal-to-noise ratio. We experimentally show a twofold improved sensitivity of our three-dimensional electrode structure to conventional planar electrodes and support these findings by finite element simulations. Finally, we report on the differentiation of polystyrene beads, primary mouse T lymphocytes and Jurkat T lymphocytes using our device.

An automated counting of living biological cells in culture populations has become an important tool for many biomedical diagnostic and research applications^1^. Currently prevalent detection schemes comprise of either optical methods such as the widely used fluorescence-activated cell sorting (FACS) technique^2^ or electronic sensing mechanisms employed by instruments using the Coulter principle. In contrast to optical methods, impedance-based sensing holds great potential for the ease of downscaling using microfluidics, parallelization and potentially label-free operation.

Such on-chip devices allow for on-site diagnostics as required for the sites of virus outbreaks in developing countries^3^. An important goal in building these minituarized particle counters is to achieve a high cell throughput while simultaneously maintaining a high sensitivity. In microfluidic impedance-sensors, the cell passes over electrodes, thus inducing a change in the impedance of the device by altering conductivity and capacitance. Typically, electrodes are either embedded on one side of the channel in a coplanar fashion^4^,^5^,^6^ or embedded on opposing sides of the channel^7^,^8^. For both approaches, the impedance measurement depends on the position of the particle in the flow^9^, where the latter design generally promises an increased tolerance to the particle position and a higher sensitivity^10^. Due to a lack of close contact between cell and electrodes, both electrode geometries suffer from leakage currents propagating through the extracellular medium. An approach to solve this problem is the use of constriction channels, whose diameter is smaller than the diameter of the cells. Currently, only rectangular channels have been used for this purpose^11^,^12^,^13^, which suffer from a low throughput and possible clogging due to an incompatibility of the rectangular cross section with the approximately circular cross section of biological cells in suspension.

A different electrode design with a reported higher sensitivity than coplanar or parallel-facing electrodes was first introduced by Martinez-Cisneros *et al*.^14^, who presented a tubular electrode structure. However, their design was limited to test frequencies in the kHz regime and their fluid flow through the microchannel was not confined to the electrode area.

Here, we present measurements and simulations of a microfluidic impedance-based flow sensor which allows both for a high measurement bandwidth by the use of radio-frequencies and displays an increased sensitivity as well as a reduced signal dependence on the particle position by the use of a tubular, rolled-up electrode geometry. A tubular coplanar waveguide (T-CPW) is embedded inside a semiconductor microtube, which does not only act as a scaffold for the electrodes but also functions as the circularly shaped microfludic channel at the sensing region with the electrodes wrapped tightly around it.

## Results and Discussion

**Figure 1:**
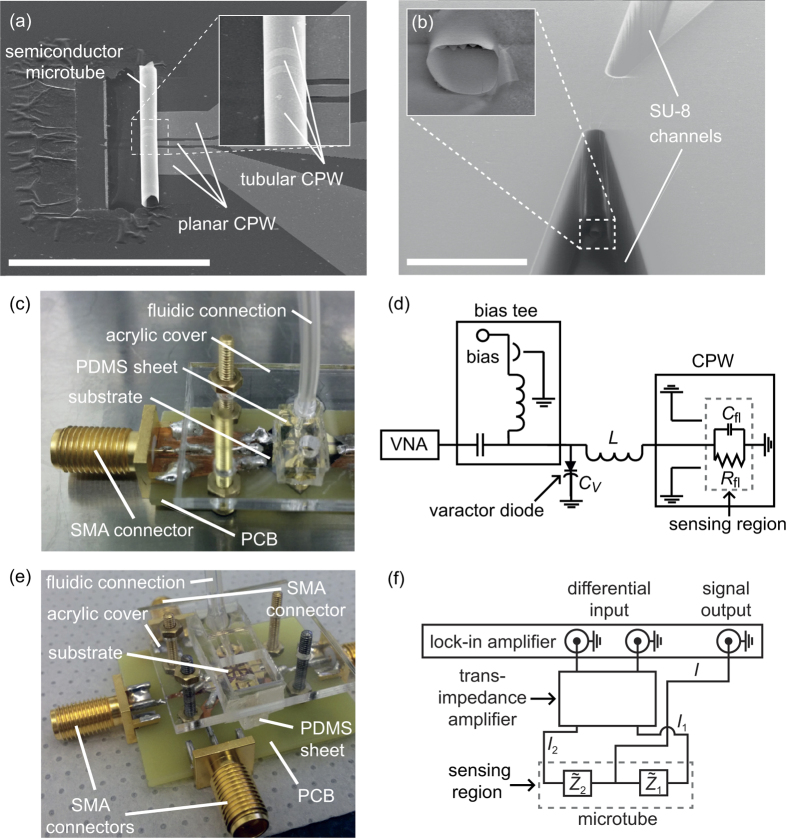
(**a**,**b**) Scanning electron microscope (SEM) images of (**a**) a rolled-up coplanar waveguide inside a GaAs/InGaAs microtube and (**b**) an SU8 channel structure for microfluidic connections to the microtube. Scalebars: 100 μm. (**c**) Photograph of the soldered microtube chip with microfluidic and SMA connections for connecting to the VNA. (**d**) Schematic of the VNA measurement circuit. (**e**) Photograph of the soldered microtube chip with microfluidic and SMA connections for connecting to the lock-in amplifier. (**f**) Schematic of the lock-in measurement circuit.

Images of a fabricated T-CPW, a T-CPW integrated into an SU-8 microchannel structure, and the sealed and electrically connected chip are shown in Fig. 1a–c,e.

### High-frequency measurements

**Figure 2:**
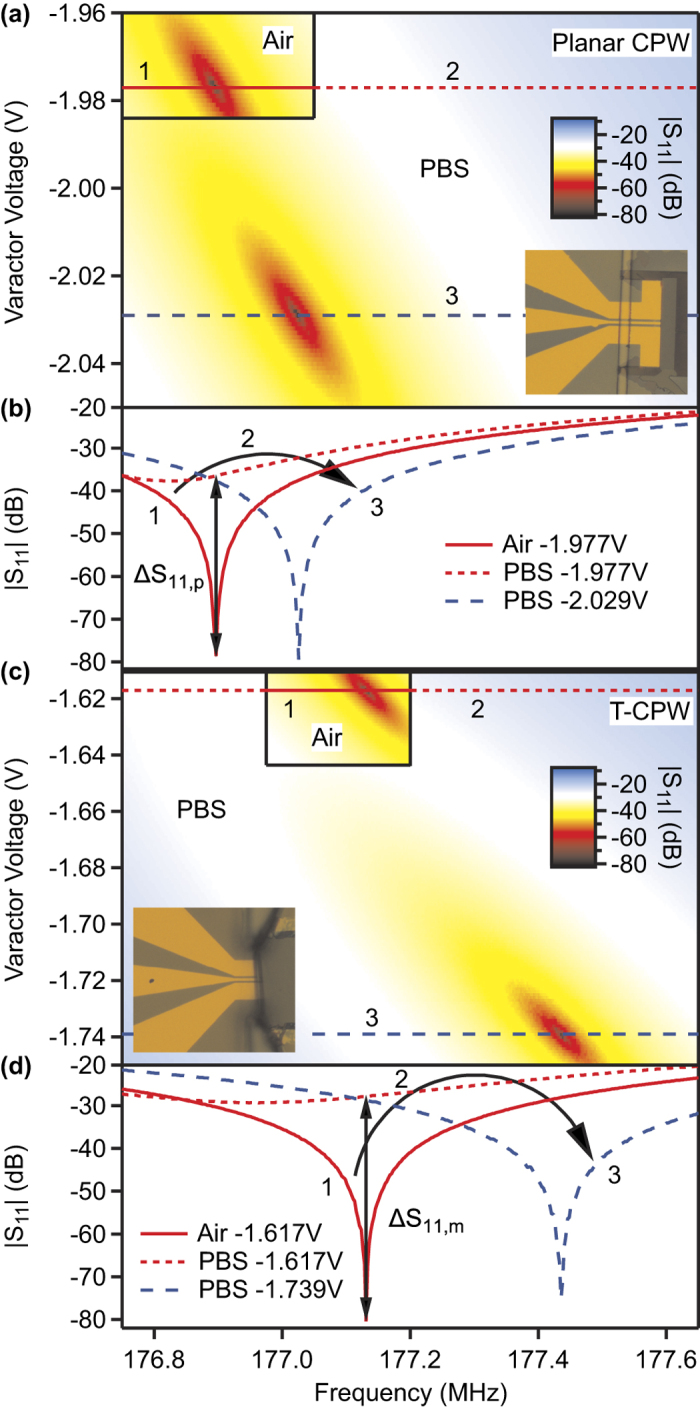
(**a**,**c**) Reflectance (S11) and varactor voltage vs. frequency and for a planar CPW and a T-CPW, which are filled with air (insets) and 1x PBS, respectively. The operating points, which mark the points with the lowest reflected signal, occur at different varactor voltages and frequencies. (**b**,**d**) Change in impedance-matched reflectance with air (solid curves labeled with 1) due to the filling of PBS into the microchannels (dotted curves labeled with 2). After filling, the device’s impedance can still be matched (dashed curves labeled with 3).

The measurement circuit is depicted in Fig. 1d. A vector network analyzer (VNA) is used for generating the electromagnetic signal and measuring its reflection from the sensing region. Details of the preparation and measurement process can be found in the Methods Section. To show the advantage of a tubular electrode structure over a simple planar structure, we built both a planar CPW as well as a T-CPW embedded inside a microtube with equal dimensions for the signal width (3 μm), the ground width (20 μm) and the distance between signal to ground (2.5 μm). An SU-8 channel with a channel width of 10 μm and a height of 9.5 μm was prepared onto the planar CPW. The microtube has a diameter of 8.5 μm. This gives cross-sectional areas of 95 μm^2^ and 57 μm^2^ for the planar-CPW microchannel and the microtube channel, respectively. The circuits are impedance-matched for air by varying the frequency and the voltage across the varactor diode while recording the *S*-parameter (see insets in Fig. 2a,c). The *S*-parameter from that varactor voltage which achieves the lowest reflection is plotted against the frequency as the solid curves in Fig. 2b,d. Then, 1x phosphate buffered saline solution (PBS) is added into the channels whereas the varactor voltage is not changed (see dotted curves in Fig. 2b,d). The linear change of the *S*-parameter at the resonance frequency amounts to Δ*S*_11,p_ = 1.5 · 10^−2^ for the planar CPW and Δ*S*_11,m_ = 4.0 · 10^−2^ for the microtube which is higher by a factor of about 2.7. This indicates an increased sensitivity for the microtube, although the sensing volume in the microtube is only about half the size. By further varying the varactor voltage (Fig. 2a,c), the impedance of the PBS filled device can then be matched to 50 Ω (see dashed curves in Fig. 2b,d) to be at the operating point even for microchannels filled with a conductive solution.

We used the microtube device as well as two devices with a planar CPW geometry where the first planar CPW I features a microchannel width of 10 μm and height of 100 μm, and the second planar CPW II a microchannel width of 10 μm and height of 10 μm to measure the flow of Jurkat T lymphocytes. The impedances of the culture-medium filled devices were matched to 50 Ω. Using the “Continuous Wave Time” (CW Time) mode of the VNA, the *S*-parameter was measured versus the time at a power of −5 dBm at an intermediate frequency bandwith of 100 kHz. By applying pressure, the cells were flushed through the devices. The cells pass through the channel although their diameter of (13.6 ± 1.2) μm is larger than the channel widths. The cell diameter was determined with a CASY cell counter.

**Figure 3:**
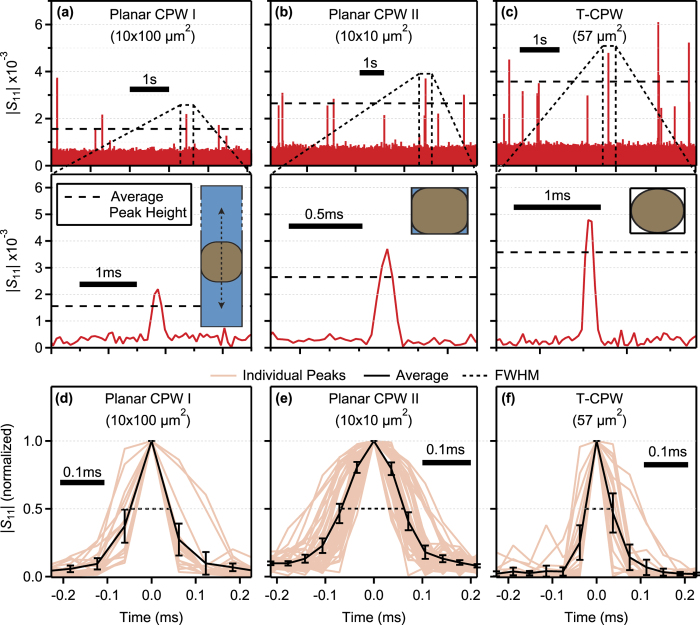
(**a**–**c**) Measurement of Jurkat T-lymphocytes flowing (**a**) over a planar CPW with microchannel width of 10 μm and height of 100 μm, (**b**) over a planar CPW with microchannel width of 10 μm and height of 10 μm, and (**c**) through a T-CPW. The insets in the lower panels show the illustrated cross section of the microchannel when a T cell passes the sensing region. Since the cross sectional area of the cells is greater than that of the microchannel, the cells are squeezed, which is indicated by their deformed shape in the illustration. (**d**–**f**) Analysis of the peak shapes. The peak heights were normalized and the resulting peaks were layed on top of each other. Red curves represent measurement data, whereas the black curves represent an average over all individual peak shapes.

An excerpt of the measurement results is shown in Fig. 3. Every time a cell passes the electrodes, an impedance mismatch occurs leading to a nonzero |*S*_11_|. The peak heights were automatically analyzed whereas only peaks with a height greater than |*S*_11_|_rms_ + 6*σ* were taken into account, where |*S*_11_|_rms_ denotes the root mean square and *σ* the standard deviation of the recorded reflection coefficient. Additionally, peaks with a width of only one measurement point were discarded to avoid detecting peaks which result from statistical noise. For the planar CPW I with the higher microchannel, the average peak height amounts to 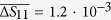 with a relative standard deviation of 43%. The planar CPW II with the smaller microchannel exhibits an average peak height of 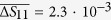 and a relative standard deviation of 28%. Finally, the average peak height for the T-CPW is the heighest at a value of 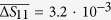, which is 2.7 times as heigh as for the planar CPW I. The relative standard deviation amounts to 44%.

The response of impedance-based flow cytometers is typically dependent on the position of the object to be detected in the microchannel and the size of the object^9^. When cells are flowing through the sensing region with a certain distance to the electrodes, less signal is being picked up. In the planar CPW I, the vertical position of the cell cannot be controlled, as depicted in the inset in the lower panel of Fig. 3a. This explains the low average peak height and the high standard deviation of this particular device. For the planar CPW II and the T-CPW, the cross sectional area of the T lymphocytes is bigger than that of the microchannels, which rules out both a positional and size dependence of the cells in the channel. Nevertheless, the standard deviation of the individual peak heights is still quite high, also for these devices. It is possible that the different signal responses of individual cells represent a different cell characteristic, e.g., that they are at different stages of apoptosis, a process which renders the plasma membrane porous and thus more conductive. This leads to a partial uptake of ions from the extracellular medium and thus, an increase of the regular cytoplasm conductivity of 0.32 S/m^15^ towards the conductivity of 1.1 S/m of the extracellular medium^16^.

**Figure 4:**
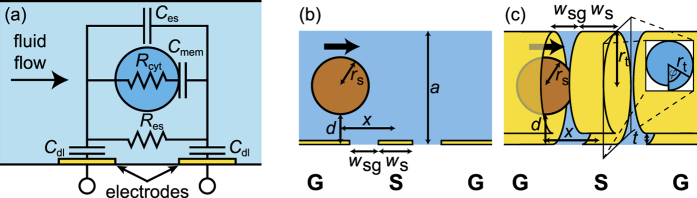
(**a**) Equivalent circuit of our impedance-based microfluidic flow sensor. (**b**,**c**) Geometry of the simulation. (**b**) depicts a planar coplanar waveguide with an electrolyte channel on top with a square-shaped cross section. The channel height is *a* = 10 μm. (**c**) depicts the geometry of a rolled-up electrode structure with an embedded tubular electrolyte channel. The microtube was parameterized as an archimedean spiral with a radius 
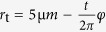
, where *t* = 0.2 μm and *φ* is the angle around the tube. The geometric parameters of the coplanar waveguide are the same for (**a**,**b**) and denote as: *w*_s_ = 3 μm and *w*_sg_ = 2.5 μm. The width of the ground conductor is 20 μm.

Remarkably, the T-CPW shows a higher variance of the peak height than the CPW II. With the aid of an equivalent circuit model as illustrated in Fig. 4a, we will show in the following how this reflects a better seal and thus lower leakage currents. Let us assume a cell population with an average cytoplasm resistance of 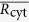 and a standard deviation of 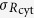 due to inherently different cell characteristics. Because of the high frequency of our electromagnetic signals, we can neglect the double layer capacitances *C*_dl_ of the electrodes^8^, as well as the plasma membrane capacitance *C*_mem_ and the capacitance through the extracellular solution *C*_es_^9^. The circuit is thus dominated by the cytoplasm resistance *R*_cyt_ and leakage currents through the extracellular solution, reflected by *R*_es_.

For the planar CPW II, one can assume the cells take on the shape as depicted in the inset of Fig. 3b. The circular cross section of the cells is incompatible to the rectangular cross section of the microchannel, leading to a not negligible *R*_es_. The overall resistance of the circuit is thus *R*_tot,CPW II_ = *R*_cyt_ · *R*_es_/(*R*_cyt_ + *R*_es_). Using error propagation, one can calculate the standard deviation of the total resistance:


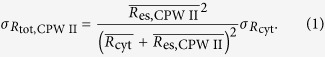


Since the fraction in Eq. 1 is less than 1, it follows that


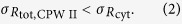


In the case of the T-CPW, the cells fill out the whole of the microtube’s cross section as depicted in the inset of Fig. 3c. Therefore, we can assume an infinite seal resistance *R*_es_, and the total resistance of the circuit writes as *R*_tot,T-CPW_ = *R*_cyt_. The standard deviation of the total resistance is thus


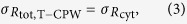


which is greater than 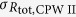, exactly what we see in the measurement. We can thus conclude that the T-CPW not only shows the greatest average peak height, it also is more sensitive to possible changes in the cell’s electric properties by eliminating leakage currents. We remark a much greater seal by using our circular microchannel approach as opposed to the use of rectangular constriction channels in other studies^11^,^12^,^13^. In typical impedance-based flow cytometric measurements, the positional and size dependence is removed by introducing a parameter called “opacity”, the ratio of the signal at a high frequency over the signal at a low frequency^7^,^9^,^17^, which imposes the need to measure at two frequencies simultaneously. In our approach, we can overcome this need and measure signals independent on size and position at a single frequency.

The cells flow over two distinct gaps between the electrodes: over the first ground electrode towards the signal electrode and then towards the second ground electrode. Since the electric field is highest between the electrodes, a double peak is expected for the change in the 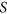-parameter^18^. An analysis of the peak shapes can be seen in Fig. 3d–f. The average peak shapes from both planar CPW and T-CPW are similar with a full width at half maximum (FWHM) of ~80 μs for the planar CPW I, ~130 μs for the CPW II, and ~60 μs for the microtube. The FWHM represents the transit time of the cells through the sensing region. The cells are thus passing through the T-CPW faster than through the other devices, indicating that it is easier for the cells to be pushed through the circular channel of the microtube than through the rectangular channels of the planar CPW device. The CPW II exhibits the slowest transition time, since the cells are clamped by the microchannel from four sides rather than from just two as in the case of the CPW I.

For both devices, no double-peak feature is discernible. On the one hand, this can be accounted for by either technical reasons, i.e., a lack of temporal resolution. Another explanation is based on physical reasons. More specifically, the size of the cells, whose diameter is greater than the distance between the two ground electrodes, causes a merging of the two peaks. By means of finite element simulations using COMSOL Multiphysics we support the latter claim and additionally investigate the performance of the T-CPW regarding particles whose diameter is smaller than that of the microtube.

### High-frequency simulations

We simulated a polystyrene sphere of varying radius with relative permittivity of *ε*_r_ = 2.5 (conductivity of 10^−4^ S/m)^4^ flowing through channels filled with electrolyte solution with a conductivity of 10 S/m. The two different geometries used can be seen in Fig. 4b,c. The geometrical parameters of the coplanar waveguide leading towards the channels are the same as in the experiment, albeit infinitely thin conductors were chosen to save simulation time. The frequency was set to 200 MHz for all simulations. The complex impedance 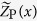 of the lumped excitation-port was recorded for different positions *x* of the polystyrene bead. From this, we calculated the *S*-parameter using


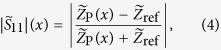


where 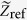 is the lumped-port impedance in the absence of a polystyrene bead. In this way, the simulated devices are numerically impedance-matched, and the magnitudes of 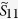 of both planar CPW and T-CPW can be compared with each other.

**Figure 5:**
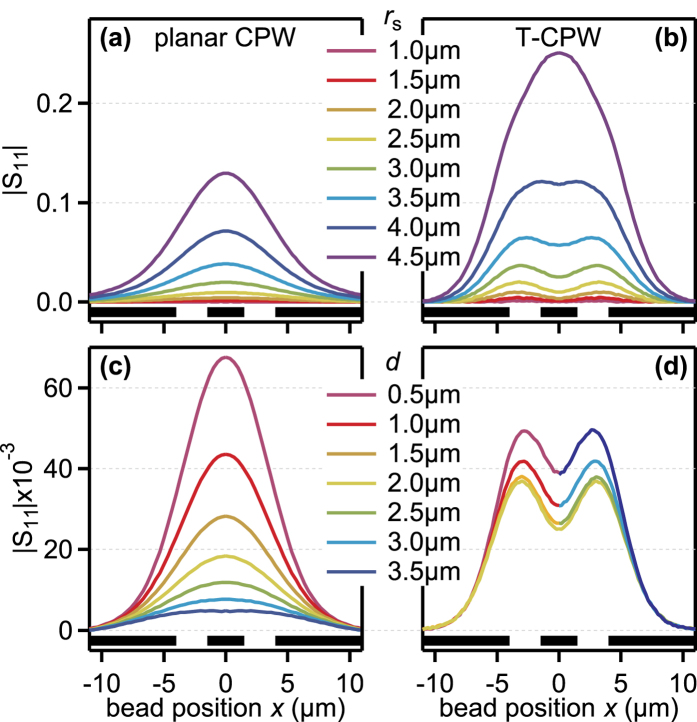
Calculated *S*-parameter response to a polystyrene bead with varying radius *r*_s_ and distance *d* from the bottom of the microchannel flowing (**a**,**c**) over a planar CPW and (**b**,**d**) through a T-CPW.

**Figure 6:**
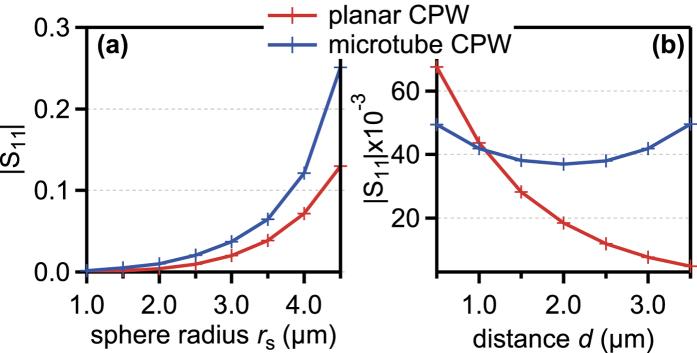
Signal levels of both planar CPW and T-CPW from Fig. 5. (**a**) depicts the dependence on the polystyrene sphere radius *r*_s_, (**b**) shows the dependence on the distance *d* of the sphere from the bottom of the microfluidic channel.

In the first parameter sweep, the sphere radius *r*_s_ was varied from 1 μm to 4.5 μm in steps of 0.5 μm, while the distance of the bead center to the floor of the microfluidic channel was kept constant at 2.5 μm, i.e., for the microtube, the sphere was kept in the middle of the channel. The result is shown in Fig. 5a,b. Generally, the peak amplitude of the microtube device is higher for the same sphere radius (see Fig. 6a). Also, the microtube exhibits double peak features for *r*_s_ < 4.5 μm, whereas for the planar CPW, only single peaks are discernible.

In the second parameter sweep, the radius of the sphere was kept constant at *r*_s_ = 3.0 μm, whereas the distance *d* of the sphere to the bottom of the microfluidic channel was varied from *d* = 0.5 μm to 3.5 μm in steps of 0.5 μm. Only for a distance of 0.5 μm, the planar CPW device shows a greater response to the bead. Also, the dependence on the position of the bead in the channel is much greater for the planar CPW. Here, the T-CPW shows a variation of only about 12% around the middle value (see Fig. 6b for a depiction of the peak amplitudes). The amplitude drops when the polystyrene bead shifts towards the middle of the tube and increases when the bead shifts towards the top of the microtube. This leads to far more consistent device responses when using the T-CPW geometry.

However, the positional dependence can not be fully eliminated. In measurements of 6 μm sized polystyrene microbeads (not shown here), we found a large relative standard deviation of 18% of the signal peak heights, which we attribute to the remaining positional dependence as evidenced by the simulations.

In short, the simulations support the findings from the measurements that the devised tubular electrode structure is much more suitable as an impedance-based cell cytometer as a planar CPW structure. It is not only generally more sensitive, it also reduces the dependence of the response of the device on the position of the sample to be detected.

### Two-frequency measurements

Lastly, we demonstrate the ability of our device to discriminate different cell types and particles. For this purpose we connect the device to a lock-in amplifier (HF2LI, Zurich Instruments). A two-frequency signal at frequencies of 12.7 Mhz and 700 kHz is applied to the center electrode of the T-CPW and is fed from the two outer electrodes via a trans-impedance amplifier into the inputs of the lock-in amplifier as shown in Fig. 1f. The two regions between the center and outer electrodes constitute two impedances 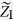 and 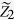, which are approximately equal when no particle is present. In this case the two currents *I*_1_ and *I*_2_ cancel out at the diffential input of the lock-in and no signal is measured. When a particle passes the electrode region, the two impedances are different and thus cause a non-zero current difference which can be measured by converting it into a voltage and amplifying with the trans-impedance amplifier.

**Figure 7:**
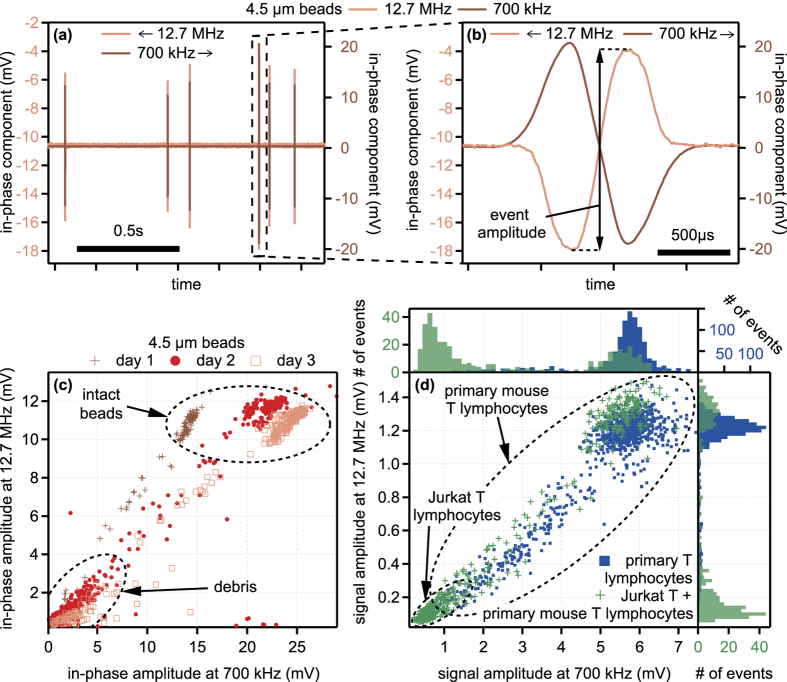
(**a**) Exemplary excerpt of the time-resolved in-phase component during the flow of 4.5 μm-sized polystyrene beads through a T-CPW device. The in-phase component was measured at two frequencies, 12.7 MHz and 700 kHz. (**b**) Detailed view of the event from panel (a). (**c**) Event amplitudes of the in-phase components of 4.5 μm-sized polystyrene beads measured at the same frequencies simultaneously. (**d**) Signal amplitudes of two different cell types measured at 12.7 MHz and 700 kHz simultaneously using a T-CPW device with a larger distance *w*_sg_ = 15 μm between center and outer electrodes. The upper and right panels show the histograms of the event amplitudes at the high and low frequency, respectively.

Polystyrene beads with a diameter of 4.5 μm were flushed through the T-CPW. An excerpt of the recorded in-phase component is shown in Fig. 7a. Every time a particle passes the sensing region, a double-peak occurs in the measurement. A close-up view of a single event is shown in the panel (b) in the figure. Note that we have two superimposed graphs for the two frequencies. The in-phase amplitudes of each event, where we define the amplitude as the maximum of the signal minus the minimum of the signal during the event, were extracted. The in-phase amplitudes at 12.7 Mhz were plotted against those at 700 kHz in Fig. 7c. The experiment was repeated three times over three consecutive days.

The signal responses can be categorized into two main areas in the diagram, the lower left and upper right area. In a control experiment, where single particles were optically examined during the measurement, the intact particles could be attributed to the upper right area. For lower signal amplitudes, no particles were discernible, i.e., the measured particles exhibited a size below the optical resolution. We therefore classify the lower left area as debris.

Over each day of measurement, the device’s response is very reproducible for the high-frequency signals, whereas we observed a shift over time towards higher signal amplitudes for the low-frequency regime. This signal increase can be attributed to an increase of the electrical double-layer capacitance, which is due to an electrochemical reaction of the gold surface with the electrolyte solution.

Additionally, we compared the T-CPW signal response of two different cell types, Jurkat T lymphocytes and primary mouse T lymphocytes. Note that the distance *w*_sg_ between center and outer electrodes of this device amounts to 15 μm, opposed to the distance of only 2.5 μm of the other device in Fig. 7c. Figure 7d shows the extracted amplitudes of the signal magnitude. First, we measured a mixture of Jurkat T lymphocytes and primary mouse T lymphocytes (green crosses in the center graph). In a second experiment, only primary mouse T lymphocytes were flushed through the T-CPW (blue squares).

Since the mixture of both cell types occupies an area in the lower left panel of the graph, which is not occupied in the measurement of only primary mouse T lymphocytes, we attribute this area to the signal of the Jurkat T lymphocytes, while we attribute the area in the top right of the center graph to the signal of the mouse T lymphocytes. We can rule out that the difference between the signal responses of the two cell types are due to the observed shift in the device’s response (see Fig. 7c) rather than due to a difference in the cells’ properties, since the signal amplitude shift over time was observed solely in the low frequency regime. However, we observe different signal amplitudes in both the low and the high frequency regime. Additionally, the localization of Jurkat T cells in the lower left corner was also verified by analyzing such cells alone (data shown in the Supplementary Fig. S1 and Fig. S2).

Although the average diameter of the Jurkat T lymphocytes is larger than that of the mouse T lymphocytes, their amplitudes are lower for both frequencies. This is particularly remarkable for the low-frequency regime, since in this regime, the size is the parameter dominating the signal amplitude^9^. A possible explanation would be the death of the cells, either elicited by too high pressures due to the squeezing of the cells through the microtube or by too high temperatures induced by the electric current. Since the pressure during the flow experiment was not set to higher values than 150 mbar, which is below a normal systolic blood pressure of 120 mmHg, we assume the cells are able to withstand these pressures.

In the following, an estimation of the temperature change is given. For one of the electric current paths from Fig. 1f, the measured signal at the lock-in amplifier is *U*_1_ ≈ 200 mV at 12.7 MHz, which, by using the feedback resistor *R*_f_ = 100 kΩ of the trans-impedance amplifier, translates to a current of


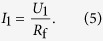


The electrical resistance *R*_fl_ along the sensing volume, i.e., the volume between the inner and outer electrodes, can be estimated as follows:


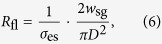


where *σ*_es_ = 1.1 S/m is the conductivity of the extracellular medium, *D* = 8.5 μm is the diameter of the microtube and *w*_sg_ is the distance between center and outer electrodes. The power loss *P* across the resistance *R*_fl_, which leads to an increase in the temperature *T* is thus given by


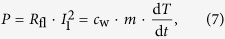


where *c*_w_ = 4.2 kJ/(kg · K) is the specific heat capacity of water, *m* is the electrolyte mass in the sensing volume and 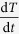 is the time derivative of the temperature. By inserting all the values, a temperature increase per sensing volume of 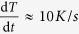 can be deduced. Note that this increase is valid only for the stationary case, i.e., no fluid flow. For the flow velocities present during our experiments of above 100 mm/s, which can be deduced from the transition times of the cells, an electrolyte volume of more than 40,000 sensing volumes per second is exchanged. This translates to a temperature increase of less than 0.3 mK of every sensing volume passing the electrodes, a negligible effect. Taken together, physical parameters, such as pressure and temperature, are unlikely to cause massive cell death during the transition through the T-CPW. Thus, it is unlikely that dead cells account for the signals.

Interestingly, primary T cells show a much broader distribution of signal amplitudes at both frequencies, see Fig. 7d. It is well known that primary T cells contain different subsets, e.g. naive, effector, memory, and regulatory T cells. Naive T cells are small in size and contain a small number of mitochondria and possibly also little endoplasmic reticulum (ER). In contrast, other T cell subsets, such as effector T cells are packed with mitochondria and ER. Thus, different cell subsets contained in primary T cells display different properties, as observed as larger cloud in the upper right corner and the more single cells stratified over a large area from upper right to lower left corner (Fig. 7d). According to its size, the large cloud in the upper right corner are naive T cells, while the other subsets are likely to be spread over a larger area, with effector T cells partially overlapping with Jurkat T cells. Since Jurkat T cells in many aspects resemble effector T cells, this is a valid assumption. The T cells in the center of Fig. 7d cannot be allocated to specific sub-type(s). In summary, we presently do not exactly know which biochemical or immunological property of the cell, e.g. size, amount of internal membranes, state of activation etc. results in what behaviour in the lumen of the T-CPW. However, this full determination requires many well-characterized T cell subtypes to be analyzed and will be subject of further investigation.

## Conclusion

We built radio-frequency impedance sensors by embedding a coplanar waveguide into a semiconductor microtube to obtain a rolled-up, tubular electrode geometry. While flowing PBS through our device and measuring the *S*-Parameter during the transition from air to PBS, this tubular geometry yields an increase in sensitivity by a factor of about 2.7 when compared to a planar electrode structure. We also demonstrated the use of this device as a highly sensitive cell counter by flowing Jurkat T lymphocytes through the microchannels. We were able to continuously flow cells through the microtubes, although their diameter is smaller than the diameter of the channel. With this method we are able to eliminate possible leakage currents. We showed an increase in average peak height by a factor of about 2.2 when compared to conventional planar electrodes. Faster measurement speeds are readily possible by increasing the intermediate frequency bandwith, albeit this simultaneously increases the noise floor.

We supported our experimental findings by means of finite element simulations. Conclusively, our device might open up a novel way of building compact, impedance-based flow sensors, which exhibit a high sensitivity even at fast measurement speeds.

Using a two-frequency signal, we demonstrated the ability of discriminating different cell types from each other, where we found lower signal amplitudes of the Jurkat T lymphocytes compared to primary mouse T lymphocytes despite their larger diameters. In the future, our increased sensitivity could be used to measure functional characteristics of T lymphocytes. We anticipate that there might be differences in the diverse T cell subclasses and differentiation stages. Likely, different phases of the cell cycle can be distinguished, since naive T lymphocytes and blasts are of different size. Also, cell death phenomena like apoptosis and necrosis might be detectable. These described changes of the cells’ electrical properties should lead to a different signature in our device response, opening a pathway of building label-free flow cytometers.

## Methods

### Device preparation

The semiconductor microtubes are built from square, 10 × 10 mm^2^ sized substrates, which consist of a heterostructure of AlAs/InGaAs/GaAs grown by molecular-beam epitaxy on top of a GaAs substrate^19^,^20^. A chromium/gold coplanar waveguide (CPW) structure is evaporated using standard photolithography techniques. Here, the chromium is used to promote the adhesion of the gold layer to the substrate. Due to a slight lattice mismatch of InGaAs and GaAs, this bilayer is strained. When the sacrificial AlAs layer is selectively etched away using 5% aqueous hydrofluoric acid solution, the top layer of InGaAs/GaAs/Cr/Au rolls up and forms a micrometer-sized tube with an embedded coplanar waveguide structure (see Fig. 1a). By choosing different layer thicknesses, the diameter of the microtubes can be precisely tuned in a range from a few hundred nanometers to tens of microns^21^. Here microtubes with diameters of about 10 μm were fabricated.

The negative photoresist SU-8 (Gersteltec GM 1075) is then spincoated on the sample. The SU-8 efficiently seals the outside surfaces of the microtube against fluid flow to ensure that liquid flows only inside the microtube^22^. Applying SU-8 on small sized and additionally, non-circular substrates leads to the formation of resist edge-beads due to the high viscosity of SU-8^23^. These edge beads cause a gap between the photo mask and the resist during exposure and thus limit the quality of the channels. Additionally, they might prevent the liquid proof sealing of the channel. In order to achieve a flat SU-8 layer, a technique for eliminating the resist edge-beads was devised. Here, after softbake at 95 °C and cooling down to a temperature of 75 °C, a silanized glass slide with an added weight is put on top of the SU-8 layer exhibiting edge beads in the order of 20 μm. At this elevated temperature, the unpolymerized SU-8 is still soft and thus, the edge beads are being pushed towards the edges of the substrate, resulting in a flat SU-8 layer. After further cooling down to room temperature, the silanization of the glass slide enables a simple removal of the glass slides from the SU-8 due to their hydrophobicity. Two SU-8 channels leading towards the microtubes’ orifices are then defined by photolithography (see Fig. 1b). The substrate is soldered onto a printed circuit board with attached SMA connectors.

We developed a technique for reversibly sealing the channels using a ~5 mm thick PDMS sheet with predrilled holes and a cover made from acrylic glass. The holes of the PDMS sheet are aligned onto the SU-8 channel structure and loosely pressed against the substrate. Then, an acrylic glass cover with CO_2_-laser drilled holes is attached over the PDMS sheet. By tightening the four screws fitted into the printed circuit board and the acrylic glass, the fluidic seal can be made to withstand pressures of over 1.7 bar. Lastly, tubes are glued into the openings of the acrylic cover for fluid filling (see Fig. 1c,e for photographs of the finished device).

### Experimental setup

The circuit diagram for the high frequency measurements is shown in Fig. 1d. A vector network analyzer (VNA, PNA 5222A, Agilent Technologies) feeds a radio-frequency electromagnetic signal to the device. Via a bias tee, a DC voltage, which controls the varactor voltage and thus the capacitance *C*_V_, is coupled into the circuit. The inductance *L* and the capacitances *C*_V_ as well as the capacitance *C*_fl_ at the end of the coplanar waveguide inside the microtube form a tank circuit with a resonance frequency. The scattering parameter 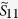, also referred to as the reflection coefficient, is given by


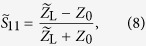


where 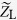 is the load impedance and *Z*_0_ = 50 Ω is the system impedance. The sensitivity for impedance measurement is highest at the resonance frequency, i.e., for 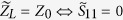. The circuit impedance 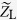 can be written as:


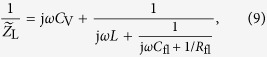


where *R*_fl_ is the resistance of the fluid inside the microtube and *ω* is the angular frequency of the electromagnetic signal. Solving 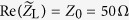 and 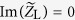 gives two equations for the frequency and the varactor voltage. Thus, by adjusting these parameters, one can match the load impedance 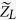 to the system impedance *Z*_0_. The result of the impedance matching is shown in Fig. 2. We obtain resonance frequencies of ~180 Mhz and high-*Q* resonance curves, with which one can get very large and sharp reflectance changes induced by only a slight change in the impedance when measuring at the impedance-matched resonance frequency.

The circuit diagram for the two-frequency measurements is shown in Fig. 1f. The signal is generated by the oscillators of the lock-in amplifier at 400 mV peak-to-peak voltage for the frequency of 12.7 Mhz and 200 mV peak-to-peak voltage for the frequency of 700 kHz. Via a trans-impedance amplifier with a feedback resistor of *R*_f_ = 100 kΩ, the current is transformed to a voltage signal, which is measured by two seperate demodulators. Measurements were carried out at sampling rates of ~230 kSamples/s at low-pass filter time-constants of 2 μs each.

### Cell culture and cell isolation

The Jurkat T lymphocytes are cultured in Roswell Park Memorial Institute (RPMI) medium 1640 with GlutaMAX^TM^, 7.5% Newborn Calf Serum (NCS) and 1.2% (w/v) penicillin/streptomycin at 37 °C in 5% CO_2_ in air. The dilution with fresh culture medium is done every two days so that the cell concentration is kept between 0.3 · 10^6^ and 1.2 · 10^6^ cells/ml.

The primary mouse T lymphocytes are isolated from the spleens of wild-type mice using the EasySep Mouse T Cell Isolation Kit (STEMCELL Technologies Inc.) following a protocol described elsewhere^24^.

## Additional Information

**How to cite this article**: Bausch, C. S. *et al*. Ultra-fast cell counters based on microtubular waveguides. *Sci. Rep.* **7**, 41584; doi: 10.1038/srep41584 (2017).

**Publisher's note:** Springer Nature remains neutral with regard to jurisdictional claims in published maps and institutional affiliations.

## Supplementary Material

Supplementary Information
