# Supplementary material for: Ultra-fast cell counters based on microtubular waveguides
Source: Sci Rep. 2017 Jan 30;7:41584. doi: 10.1038/srep41584 (PMC5278506; doi:10.1038/srep41584)
Supplement: Supplementary Information [file srep41584-s1.pdf]

Supplementary Information

Ultra-fast cell counters based on microtubular waveguides

Cornelius S. Bausch[1,2,*], Christian Heyn[1], Wolfgang Hansen[1], Insa Wolf[3], Björn-Philipp Diercks[3], Andreas H. Guse[3], and Robert H. Blick[1,2]

[1]Institute of Nanostructure and Solid State Physics, University of Hamburg, Jungiusstraße 11c, Hamburg, Germany
[2]Center for Hybrid Nanostructures, University of Hamburg, Falkenried 88, Hamburg, Germany
[3]Calcium Signaling Group, Department of Biochemistry and Molecular Cell Biology, University Medical Center Hamburg-Eppendorf, Martinistraße 52, Hamburg, Germany
[*]cbausch@physnet.uni-hamburg.de

This Supplementary Information contains measurement data from additional devices with differing electrode geometries. Figure S1 shows measurements from a device with a distance of $w_{sg} = 2.5\,\mu m$ between the center and outer electrodes, while Fig. S2 features measurement data from a device with a distance of $w_{sg} = 10\,\mu m$. The measurement data depicted in Fig. S1 was recorded by first flushing primary mouse T lymphocytes, and then Jurkat T lymphocytes, both suspended in RPMI 1640, through the device. On the other hand, the measurement data depicted in Fig. S2 was recorded by first flushing a mixture of both primary mouse T lymphocytes and Jurkat T lymphocytes, and then, only Jurkat T lymphocytes through the device. Both measurements confirm that the device responses from Jurkat T lymphocytes exhibit lower amplitudes than from primary mouse T lymphocytes.

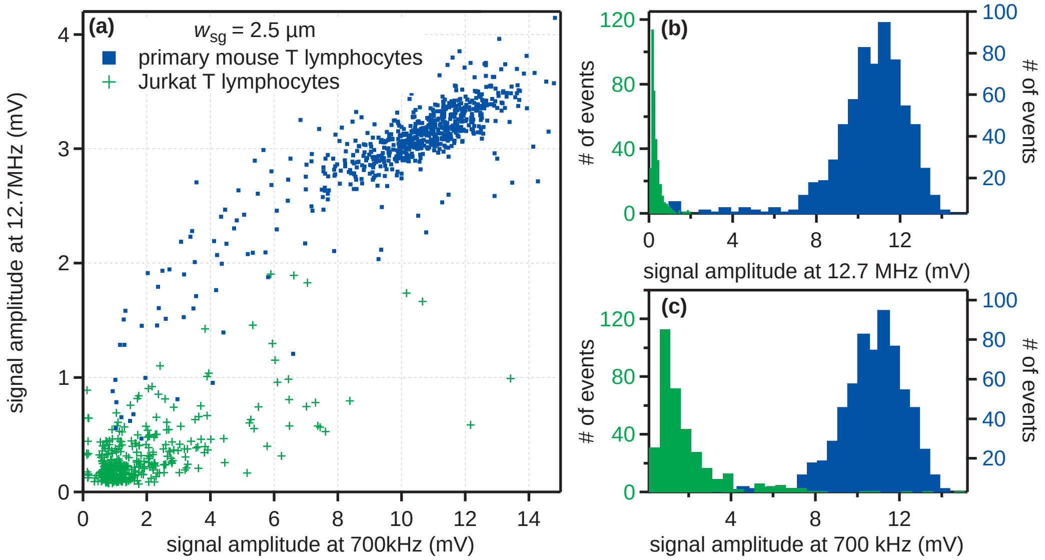

Figure S1. (a) Signal amplitudes of two different cell types measured at 12.7MHz and 700kHz simultaneously using a T-CPW device with a distance of $w_{sg} = 2.5\,\mu m$ between center and outer electrodes. (b,c) Histograms of the event amplitudes from (a) at the high and low frequency, respectively.

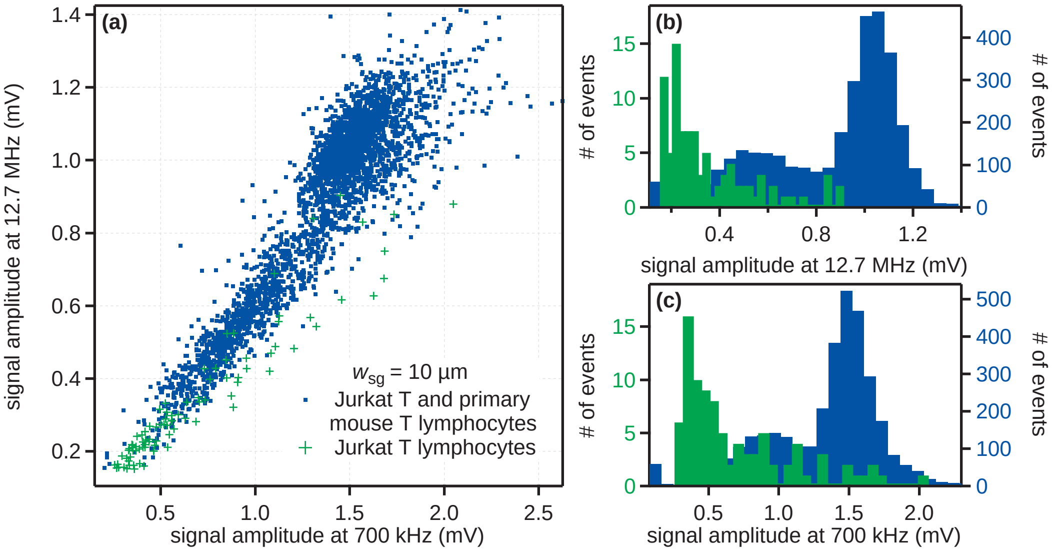

Figure S2. (a) Signal amplitudes of two different cell types measured at 12.7MHz and 700kHz simultaneously using a T-CPW device with a distance of $w_{sg} = 10\,\mu$m between center and outer electrodes. (b,c) Histograms of the event amplitudes from (a) at the high and low frequency, respectively.
